# Understanding bicycling in cities using system dynamics modelling

**DOI:** 10.1016/j.jth.2017.08.002

**Published:** 2017-12

**Authors:** Alexandra Macmillan, James Woodcock

**Affiliations:** a Department of Preventive and Social Medicine, University of Otago, PO Box 56, Dunedin 9054, New Zealand; b Centre for Diet and Activity Research (CEDAR), University of Cambridge, Box 285 Institute of Metabolic Science, Cambridge, CB2 0QQ, UK

## Abstract

**Background:**

Increasing urban bicycling has established net benefits for human and environmental health. Questions remain about which policies are needed and in what order, to achieve an increase in cycling while avoiding negative consequences. Novel ways of considering cycling policy are needed, bringing together expertise across policy, community and research to develop a shared understanding of the dynamically complex cycling system. In this paper we use a collaborative learning process to develop a dynamic causal model of urban cycling to develop consensus about the nature and order of policies needed in different cycling contexts to optimise outcomes.

**Methods:**

We used participatory system dynamics modelling to develop causal loop diagrams (CLDs) of cycling in three contrasting contexts: Auckland, London and Nijmegen. We combined qualitative interviews and workshops to develop the CLDs. We used the three CLDs to compare and contrast influences on cycling at different points on a “cycling trajectory” and drew out policy insights.

**Results:**

The three CLDs consisted of feedback loops dynamically influencing cycling, with significant overlap between the three diagrams. Common reinforcing patterns emerged: growing numbers of people cycling lifts political will to improve the environment; cycling safety in numbers drives further growth; and more cycling can lead to normalisation across the population. By contrast, limits to growth varied as cycling increases. In Auckland and London, real and perceived danger was considered the main limit, with added barriers to normalisation in London. Cycling congestion and “market saturation” were important in the Netherlands.

**Conclusions:**

A generalisable, dynamic causal theory for urban cycling enables a more ordered set of policy recommendations for different cities on a cycling trajectory. Participation meant the collective knowledge of cycling stakeholders was represented and triangulated with research evidence. Extending this research to further cities, especially in low-middle income countries, would enhance generalizability of the CLDs.

## Highlights

• Increasing cycling in cities is good for health and addressing climate change.
• We used participatory system dynamics modelling to understand urban cycling.
• Feedback loops help determine policies for different cycling contexts.
• A generalisable causal theory of cycling can enable context specific ordered policies.
• Participation made collaborative learning across stakeholders possible.

## 1 Introduction

Increasing urban bicycling as a transport mode in cities has established net benefits for human health across a range of social, physical and mental outcomes (de Hartog et al., 2010, Woodcock et al., 2013, Woodcock et al., 2009, Lindsay et al., 2011, Macmillan et al., 2014). These include increasing physical activity, enhanced neighbourhood social connection and fairer, low-cost access to health promoting education, employment, goods and services. In addition, when bicycling replaces motor vehicle use for transport trips, there is significant potential to decrease transport's contribution to climate change, air pollution, and road traffic injury.

Previous research to understand cycling in cities represents a body of disparate evidence about influences and outcomes. Analysis of travel data has contributed to an understanding of individual factors that are associated with cycling, such as age, gender and socioeconomic status (Walking, 2013, Steinbach et al., 2011, Yang et al., 2010). Survey research has concentrated on perceived barriers to cycling, including fear of injury, trip distance, weather and topography (Daley et al., 2008, Fishman et al., 2014, Goldsmith, 1992, Winters et al., 2010, Parkin et al., 2007). Significant weight in research has also been given to the role of behaviour change programmes in promoting cycling, in contrast to changing the cycling environment (Yang et al., 2010). More recently, a body of research is emerging based on natural experiments to understand environmental factors that influence individuals cycling (Goodman et al., 2014, Wardlaw, 2014, Sahlqvist et al., 2015, Heinen et al., 2015, Keall et al., 2015, Goodman et al., 2013). These studies have demonstrated modest increases in cycling from small-scale infrastructure interventions. Overall, it can be concluded from this body of evidence that high quality infrastructure may be a promising route to achieving mass cycling, while behavioural interventions alone are unlikely to achieve sustained cycling growth. Establishing robust epidemiological evidence about the effectiveness of interventions to improve and encourage cycling is limited by methodological difficulties and expense, reinforcing the importance of modelling for understanding future implications of cycling policies (Macmillan et al., 2013, Mulvaney et al., 2015).

Perhaps as a result of these disparate sources of evidence, there is disagreement amongst transport decision-makers about how to change the shape of trends in cycling (e.g. from decline into growth) and achieve a sustained growth in cycling, whether the context is a car-dependent city with very low levels of cycling, or a city where bicycling is already a major mode of transport. For example, in the Australian National Cycling Strategy (Austroads, 2010), cycling promotion is the first priority, while in New Zealand the top priority is investment in urban cycling infrastructure to improve cycling safety (Transport Agency, 2015), despite these countries having similar cycling mode shares and urban environments. There is evidence of policy uncertainty about the relative importance of behaviour change interventions; targeting; investment in cycling-specific infrastructure; and the role of land use and urban design (Wardlaw, 2014, Noland and Kunreuther, 1995, Buehler and Pucher, 2011, Pucher and Buehler, 2008). Furthermore, the above examples demonstrate there is debate about the order of policy implementation to successfully achieve sustained growth in bicycling.

Procedural issues also make an effective transition to cycling growth more difficult, particularly in cities where policies that support motor vehicle use are dominant. Transport policy-making, on the whole, continues to be characterised by technocratic processes and strong interests vested in the status quo, with little meaningful collective input from wider stakeholders (including “would-be” cyclists) to understand the complex influences on transport patterns or debate pathways for reaching desired outcomes of policy (Bickerstaff et al., 2002, Booth and Richardson, 2001, McAndrews and Marcus, 2015, Bickerstaff and Walker, 2005).

The complexity of cycling as a policy issue, uncertainty about policy effectiveness and procedural issues in transport policy all suggest that novel ways of considering cycling policy are needed. We suggest these should synthesise expertise from policy, community (including existing and “would-be” cyclists) and research stakeholders to develop a shared understanding of cycling, reflecting recommendations from research about decision-making in complex areas such as urban planning for health and sustainability (Macmillan et al., 2014, Bickerstaff and Walker, 2005, Beall and Ford, 2010, van den Belt, 2004, Vennix, 1999). As has previously been argued, methods should also aim to incorporate the dynamic complexity of influences and outcomes that determine trends in cycling (Macmillan et al., 2014). In this paper, we use participatory system dynamics (SD) modelling to address these evidential and procedural challenges. Participatory SD modelling involves a range of stakeholders in a collaborative learning process to develop a shared theory about the causes of trends over time in a complex system, and the policies that are likely to have a desired influence on observed trends (Beall and Ford, 2010, van den Belt, 2004, Vennix, 1999).

In high income countries of the global west, we postulate that four groups of cities or countries may be placed at different points on a theoretical trajectory towards cycling being a common mode of transport: a group where cycling is already a widely used mode, with a vision to further increase; a group where cycling has been growing and contributes between 5% and 10% of all trips, a group where cycling has seen a small amount of growth and is between 1% and 5% of trips, and a group where cycling is almost non-existent (around 1% of all trips) and has been that way for a significant period of time. It is likely that different influences take prominence at different places along this trajectory and therefore the most effective policies will vary.

A dynamic causal theory about cycling has previously been developed using participatory SD modelling in Auckland, New Zealand, a city with longstanding low levels of cycling and high levels of car use. This theory centralised cycling injury and perception of safety to explain the main influences on cycling over time. However, it is unclear whether the insights developed in this research can be generalised to other cities.

The aim of this research was therefore to use a collaborative learning process to build on the initial causal model for cycling developed in Auckland. We aimed to test the generalizability of the causal model for cities in the groups described above, and to enhance understanding of the system across stakeholder groups. By building consensus across cities about the causal theory, we aimed to develop agreement about the effective policies for achieving a sustained increase in urban cycling for transport while simultaneously benefiting health and environmental outcomes.

## 2 Methods

### 2.1 Participatory system dynamics (SD) modelling

We used participatory SD modelling to elicit a qualitative causal model of the influences and outcomes of cycling. We based this research on the following SD modelling principles (Forrester, 1969, Forrester, 1980, Sterman, 2006, Sterman, 2000, Richardson, 2011).

1. Complex systems include many interacting variables that change over time
2. Interaction between variables is characterised by reinforcing (positive) and balancing (negative) feedback loops and non-linear relationships
3. Patterns of interaction within feedback loops explain system behaviour over time
4. Complex systems are also characterised by the accumulation of “stocks”: variables with a measurable value at any point in time, e.g. people, information, or material resources
5. Time is an important component of complex systems and relationships may change variables at different rates over time, creating tensions between short- and long-term policy effects

While many SD modelling endeavours are undertaken by groups of researchers or technicians, participatory SD modelling explicitly includes a wide range of stakeholders, and is often focused on public policy problems. It has been successfully used to improve decision making in a variety of relevant disciplines, including urban planning, transport policy, road safety and public health. The method has also been used to consider the outcomes of transport policies on air quality (Stave, 2002) and understand the costs and benefits of cycling policies (Macmillan et al., 2014). As with many SD modelling efforts, these examples aimed to provide insights about future dynamic effects of policy alternatives by relating them to the system structure, as opposed to providing point predictions about outcomes at a future time. In the context of urban cycling, participatory SD provides an opportunity to bring together disparate sources of evidence with the understanding of policy makers, practitioners, and advocacy groups. It can also potentially support policy makers who typically face major challenges in implementing change by enabling them to communicate more confidently about desired and expected outcomes across a range of domains of interest.

Saeed (1992) describes a useful generalisable method for an SDM process that uses repeated cycles, starting with desired outcomes, then moving through the following stages: understanding of problem trends related to these outcomes; qualitative representation of the system structure; development of a dynamic simulation model; scenario experimentation; and policy design. This paper describes the first part of such a process, namely the development of an initial shared qualitative system understanding of urban cycling.

### 2.2 Previous development of the Auckland causal loop diagram

**Fig. 1:**
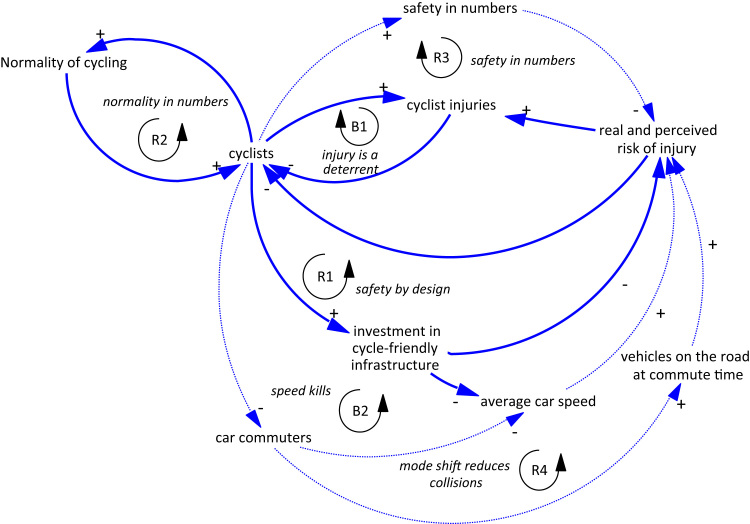
Causal loop diagram for commuter cycling in Auckland (Macmillan et al., 2014). Variables in boxes are those whose levels we are interested in following over time (stocks). Arrows with positive signs (+) indicate that a change in the arrow-tail variable leads to a corresponding change in the arrow-head variable. Arrows with a negative sign (-) indicate that a change in the arrow-tail variable leads to an inverse change in the arrow-head variable. R – Reinforcing loop, the result of which is an amplification of the initial pattern of behaviour. B – Balancing loop, the result of which is a dampening of the initial pattern of behaviour). The dashed connection was one where there remained significant uncertainty about the current existence of the relationship. This diagram has been reproduced with permission from http://ehp.niehs.nih.gov/1307250/.

In this research we used a combination of primary (interview and workshop) and secondary (statistics and research) data to develop a qualitative set of feedback loops, known as a causal loop diagram (CLD), to describe a shared dynamic causal theory about what determines trends in urban cycling. The development of a CLD can be seen to correspond to the qualitative theoretical approach known as “constructivist grounded theory” (Charmaz, 2005), where inductive analysis of qualitative data is undertaken to develop a theory about underlying sociocultural, physical and technological structures (Braun and Clarke, 2006). We used the CLD developed in Auckland as the starting point for qualitative work in London, UK and the Netherlands (Fig. 1).

The participatory process used to develop the Auckland CLD has been previously described (Macmillan et al., 2014). The Auckland CLD includes three feedback loops that were considered to be active and three “potential” loops that could be activated by an increase in cycling. These feedback loops incorporate both structural and behavioural aspects of cycling (Gatersleben and Appleton, 2007). They are described below with supporting evidence from the literature:

B1 *injury is a deterrent.* This balancing loop was considered the most important feedback in Auckland. In the absence of safe environments, more cyclists leads to more injuries, which increases fear of injury, deterring people and dampening further growth in cycling. Fear of injury is reported as a strong deterrent to cycling (Daley et al., 2008, Winters et al., 2010, Kingham et al., 2011, Pooley et al., 2010). On its own, this loop would lead to a low oscillating trend in cycling over time.

R1 *safety by design.* More people cycling results in greater advocacy for improved conditions, which in turn can improve actual and perceived safety, attracting further growth in cycling.

R2 *normality in numbers.* More people cycling tends to mean a broader range of cycling by gender, ethnicity and age, and also tends to mean a wider range of bicycles and gear. Together, these factors lead to an improved perception of cycling as a socially acceptable, normal part of everyday life, encouraging more people to cycling in a self-reinforcing way (Pucher and Buehler, 2008).

Three further loops were considered possible at higher levels of cycling:

R3 *safety in numbers.* A widely acknowledged (but poorly understood) phenomenon in the road safety literature is the reduction in risk that occurs with increasing mode share. More cyclists can mean less risk of injury per cyclist, a consequent improvement in perception of safety and therefore a reinforcing pattern encouraging further cycling growth. There is some poor quality and cross-sectional evidence to support this (Jacobsen, 2003, Tin Tin et al., 2011, Vandenbulcke et al., 2009), although the strength of the effect seen in these studies likely combines direct effects of cyclist numbers on driver behaviours, and the effects of infrastructure on safety and therefore cyclist numbers (“numbers in safety”) effects (Bhatia and Wier, 2011, Wegman et al., 2010, Elvik and Bjørnskau, 2016).

R4 *mode shift reduces collisions*. A significant shift away from car use to cycling would result in fewer vehicles and therefore lower risk of collision, as well as lower traffic volumes feeling safer for potential cyclists. As a consequence, further cycling would be encouraged (Turner et al., 2009).

B2 *speed kills*. If a significant mode shift was achieved without reallocation of existing road space away from motor vehicles, there is some concern that less congestion would increase motor vehicle speeds, undermining improvements in actual and perceived safety and dampening growth in cycling. However, there is little evidence that this occurs on urban roads (Quddus and Wang, 2009).

In this study we aimed to strengthen the validity of our causal theory about cycling in cities and understand the generalisability of both the causal theory and policy recommendations by repeating the first three parts of the generalized heuristic in two further, contrasting cycling contexts.

### 2.3 London

In London, we used a purposive sampling strategy based on an *a priori* sampling frame to identify stakeholders with an interest in London cycling policy, aiming for a group of 20–30 representatives. The sampling frame included government (Transport for London and UK Department for Transport), research, community advocacy, health sector organisations and transport engineers and design consultants. We recruited participants by direct contact with pre-determined organisations in each of the groups in the sampling frame, as well as through the suggestions of participants.

We met individually to discuss the project with a subset of initial participants, with the aim of establishing whether the Auckland CLD could be used as the basis for participatory SD modelling in London. In addition, we presented the Auckland CLDs for discussion at an interactive workshop during the 2012 national Active Travel Conference in Leicester, UK (Macmillan and Woodcock, 2012).

These discussions revealed enough similarities between stakeholder understandings in London and Auckland to give us confidence in the Auckland CLD as the basis for workshop discussions in London rather than starting again at the interview stage. However, the discussions also indicated that more nuanced feedbacks were considered to be occurring in London relating to cycling normality, safety in numbers and advocacy for investment because of the recent growth in cycling uptake in London. In particular, an improved understanding of safety in numbers for cycling was seen as a priority. We therefore convened a specific workshop with a specific set of stakeholders covering research, policy and advocacy interests in safety in numbers. This involved a presentation of the research evidence about safety in numbers, followed by discussion about a proposed causal theory for safety in numbers based on the literature.

Using the Auckland CLD, as well as detailed feedback from the meetings, we developed a refined set of feedback loops summarising the shared causal theory about cycling in London, across a number of sub-system sector CLDs. This group of sector CLDs was discussed and refined at a further 3-hour stakeholder workshop, involving stakeholders with a broader interest in cycling. The workshop began with presentations to provide a background to the project; data about trends, hopes and concerns for cycling and cycling safety in London; and an introduction to the principles and language of system dynamics modelling. Participants were then allocated to small groups mixing organisational roles. These groups rotated through facilitated discussions about each of the CLDs. Following a description of the feedback loops by the facilitator, each group was asked to discuss whether there were feedbacks that they disagreed with or that resonated with their understanding; any loops or connections that were missing; and whether there were loops that might be acting more strongly than others currently in London to explain the trends over time in cycling. Finally, groups were asked to identify any useful sources of data about relationships. Each group was encouraged to write and draw on the diagram and facilitators took notes of the discussions. Each group had the opportunity to review and discuss every CLD.

Following the workshop, we used the facilitators’ notes, verbal comments and the edited diagrams to refine the set of CLDs. Refinements were made to the preliminary feedbacks reflecting the comments and debate in the workshop, as well as triangulating the data from the workshops and discussions with the multidisciplinary literatures about cycling. Unresolved areas of debate and conflicting theories of causality are shown in these refined loops. We circulated a shared “working version” of the CLDs with a narrative description for further feedback, as well as for their use in discussing future policy options.

### 2.4 Netherlands

Working closely with system dynamics colleagues at Radboud University in Nijmegen, we used the same sampling frame to invite cycling policy stakeholders from across cities in the Netherlands to a single half-day workshop in Nijmegen. System dynamics postgraduate students acted both as participants and group facilitators, since they had both community stakeholder experiences of cycling, as well as SD skills. Cycling already has a high mode share of transport trips across the Netherlands and we were able to include stakeholders at a national level from across the country. However, we were less confident that the CLDs already developed in low cycling contexts would be transferrable to urban cycling in the Netherlands, so we designed the workshop to develop a new set of CLDs based entirely on the perceptions of the participants.

The afternoon included five large and small group exercises to: identify the main trends over time of importance for cycling in the Netherlands, including future hopes and concerns; understand influences on cycling and outcomes to help develop feedback loops; small group work to develop feedback loops; bringing the feedback loops together into a shared CLD with opportunities for disagreement and debate; identify policy insights for the Netherlands. Finally, we aimed to discuss similarities and important differences between the CLDs from Auckland, London and the Netherlands; and discuss whether policy priorities could be identified for different points on a possible generalisable “trajectory” of urban cycling.

Following the workshop, notes, workshop diagrams and facilitator comments were used to refine an electronic CLD for urban cycling in the Netherlands. This CLD, a descriptive narrative and policy insights were combined into a report for the participants to use in their cycling policy roles.

## 3 Results

### 3.1 London

Initial discussions with London stakeholders indicated that potentially nuanced feedbacks were occurring in London relating to cycling normality and advocacy for investment. Fifteen participants attended a workshop was to specifically discuss cycling safety in numbers (6 March 2013). Following the development of preliminary feedback loops for London, 32 people attended the review workshop in May 2013 (20 men and 12 women): 12 people who identified themselves as cycling advocates; 4 policy makers across health and transport; 10 academics working in public health, transport and policy studies; 5 transport engineers and planners working as consultants; and 1 NHS manager.

**Fig. 2:**
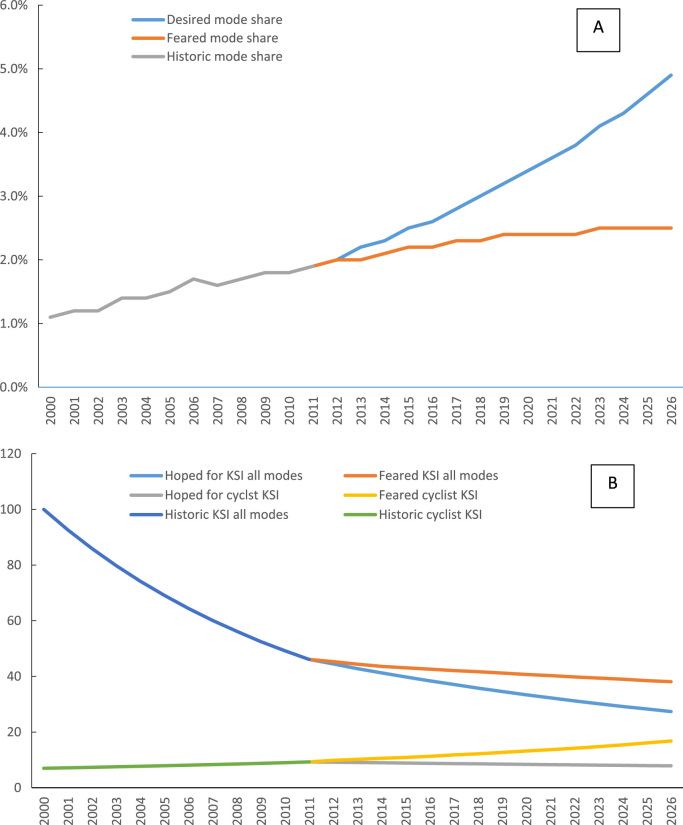
Historical, desired and feared trends in London cycling. Graph A shows trends in cycling mode share as the percentage of all trips. Graph B shows trends in all road traffic and cycling killed and seriously injured people (KSI).

Past trends in cycling in London, as well as the collective desired and feared trends over time in cycling are shown in Fig. 2. Stakeholders considered two trends important. Fig. 2a demonstrates trends over time in the mode share of cycling in London (percentage of all trips). London has seen an increase in cycling in recent times, with cycling having doubled in the past 10 years. The 2013 Mayor's vision for cycling set targets for a further increase to a 5% modal share by 2026, with the GLA Transport Committee calling for a more ambitious target of 10%. However, growth in London cycling has been spatially and demographically uneven. Much of the increase has been seen in inner London, particularly in commuting from inner to central London. The people who have newly taken up cycling in London are more likely to be male, younger to middle aged and white (Walking, 2013). Although cross-sectional studies suggest there is a strong association between higher cycling rates and more gender and age equitable cycling in areas where cycling has increased, the gender ratio in London has not improved significantly and the age ratio has worsened (Aldred et al., 2015). For the future, there is concern that the increase in cycling is being dampened by concerns about safety, with perception of safety being the major barrier to new and increased cycling (Transport for London 2012). Fig. 2b therefore explores past and future trends in overall road traffic injuries and those specific to cycling, combining numbers killed and seriously injured (KSI). In recent times, growth in cycling has been accompanied by a growth in serious injuries (STATS19, 2013, Transport for London, 2013). Furthermore, at that time it appeared that the absolute risk for cyclists of serious injuries had not fallen since 2004, while cyclists make up an increasing proportion of road traffic deaths and injuries (KSI) (Transport for London, 2013). There was concern that in the future increasing cycling may therefore make it more difficult to meet the overall road traffic injury target of a 40% reduction in KSI by 2040 (Transport for London, 2013). On the other hand, the desired future is for KSI amongst cyclists to stabilise and for total KSI to fall.

**Fig. 3:**
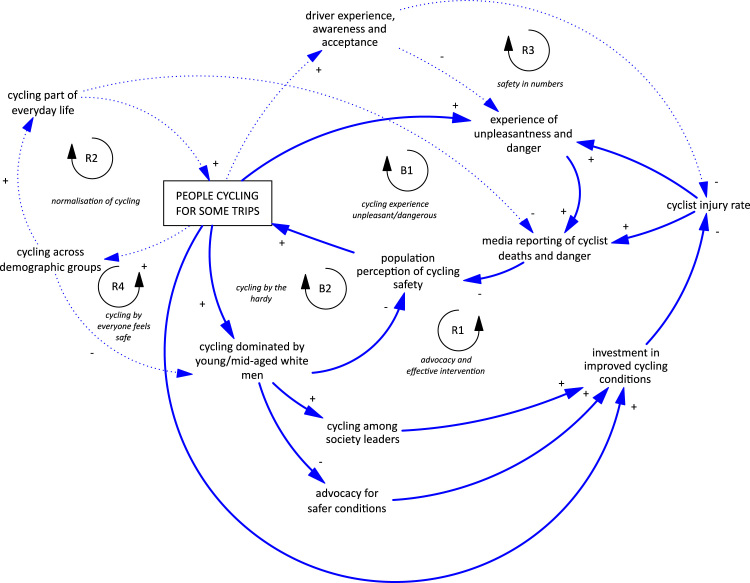
Causal loop diagram for cycling in London.

An overview of the CLDs resulting from the London workshops is shown in Fig. 3. Although it is in many ways similar to the Auckland CLD, the London diagram has some important points of difference and provides further insight into some of the underlying mechanisms for the same loops. In addition, the London stakeholders were able to discuss the cycling CLD in more depth, developing more detailed, nuanced CLDs for each of the feedback loops summarised by the overview. This reflected both the specific focus on cycling and the experience of increasing cycling in London that has not yet been observed in Auckland. These more detailed diagrams were helpful for opening up discussion and debate where we began with the CLD from Auckland, reflected the recent growth in cycling that has been seen in London, as well as reflecting the complex nature of that growth across the city and by particular parts of the population. The demographics of people cycling was considered to be playing an important role in cycling normalisation, stigmatisation and advocacy. In the London CLD, actual injuries and perception of cycling safety are separate. Six feedback loops summarise cycling in London, although only four were considered to be currently active. All six are described below and the more detailed CLDs are available in the Supplemental material.

B1 *cycling experience unpleasant/dangerous.* This is very similar to the main balancing loop in the Auckland CLD, with the added insight that not only do experienced injuries and near misses dampen growth, but also the impact of increased injuries is mediated through media reporting (Macmillan et al., 2016). In addition, there were other unpleasant experiences of cycling that were thought to be putting people off, including acts of aggression by drivers and close calls, or “almost” injuries, where cyclists experience near misses with motor vehicles (Aldred and Crosweller, 2015). Although this loop was considered to be very important in London, some helpful reinforcing loops were considered also to be active.

R1 *advocacy and effective intervention.* This loop is very similar to the R1 loop in the Auckland CLD, with an overall reinforcing pattern of behaviour resulting from more cyclists across the population advocating for better conditions. However, stakeholders considered there to be conflicting mechanisms at play in London. Early increases in cycling have been dominated by a reasonably privileged group of white men (Walking, 2013). This group may dominate because they are more tolerant of adverse cycling conditions, cycle at higher speeds and therefore experience less danger (Aldred and Crosweller, 2015), are in a privileged position (and therefore tolerant to stigma) and take journeys for which cycling is most advantageous (commuting towards central London due to congestion on roads, overcrowded public transport, and lack of car parking). For these reasons, they may be less likely to advocate for infrastructure and conditions that will make cycling safer and more attractive across the population. Conversely, this group also includes social leaders (such as London's Mayor at that time and prominent journalists) who are in a strong position to influence change when they do choose to advocate. On balance, this helpful reinforcing effect across the population was considered to dominate.

R2 *normalisation of cycling.* This is the same reinforcing loop as seen in Auckland, although it was considered to be more active in London and consequently more nuanced, with stigmatisation also a playing an important role because of the dominance of cycling by a particular sector of the population.

B2 *cycling by the hardy.* As described above, an early increase in cycling has largely been dominated by relatively risk-tolerant men cycling into central London. Despite their helpful advocacy impact (R1), it was thought that their continued dominance undermines the perception of safety of cycling more generally and therefore acts to dampen a further increase in cycling across the population.

R3 *cycling by everyone feels safe.* If cycling across demographic groups increases sufficiently in the future, it could counteract the B2 loop above. This would turn B2 into a helpful reinforcing loop by improving the population's perception of cycling safety.

R4 *safety in numbers*. This loop is the same as seen in the Auckland CLD. Stakeholders provided detailed insight into the complex mechanisms underpinning this overall reinforcing loop, emphasising that early increases in cycling may aggravate existing tensions between people cycling and people driving (see B1) before more helpful reinforcing mechanisms take over.

### 3.2 Netherlands

The single cycling system dynamics workshop was held in October 2013 at Radboud University with 24 participants, including 12 Dutch cycling stakeholders, two group model building SD experts and 12 students of the European Masters in SD. The group included seven women, all of whom were Masters students. The cycling stakeholders included six transport consultants from around the country, three local and national cycling advocates, a city councillor, one cycle courier and one spatial planning academic with a special interest in cycling. All the cycling stakeholders were men, while seven of the system dynamics modelling participants were women. The students were significantly younger and represented a wide range of nationalities. Most of them had come to Nijmegen to study from their home countries and had therefore lived in the Netherlands for a relatively short period of time.

The combination of SDM and cycling expertise allowed the participants to rapidly combine content and methodological knowledge to come up with an initial qualitative model.

Participants undertook seven main tasks followed by reflection and evaluation:

1. Individual expression of specific policy priorities for the Netherlands
2. Group development of influences and outcomes list related to cycling in Dutch cities
3. Small group development of feedback loops
4. Assimilation of all feedback loops into a single CLD
5. Comparison of CLDs across the three case studies and discussion about the role of the media
6. Insights about policies over a trajectory towards more cycling
7. Policy insights for the Netherlands

The stakeholders collectively told a story of decline in cycling after the 1950s, with the advent of cheap cars and fuel. This trend was reversed in the 1980s, with a concerted effort to revitalise cycling in Dutch cities. However, more recently it was considered that cycling's mode share has stopped growing. There was a shared desire for cycling's mode share to continue to increase to levels seen in the 1950s. There was also a desire to see the use of electric bikes grow to support the overall growth in the face of lengthening trip distances. On the other hand, stakeholders were concerned that the mode share of cycling would not grow, or even perhaps decline again. The discussions about this were particularly focused on children's safety, parental concerns and cycling to school and participants were concerned that this decline would continue as more parents take their children to school in the car. However, this perception of decline has not yet been seen in aggregate Dutch data, which suggests that cycling is continuing to increase across age groups (Kennisinstituut voor Mobiliteitsbeleid (KiM), 2016).

**Fig. 4:**
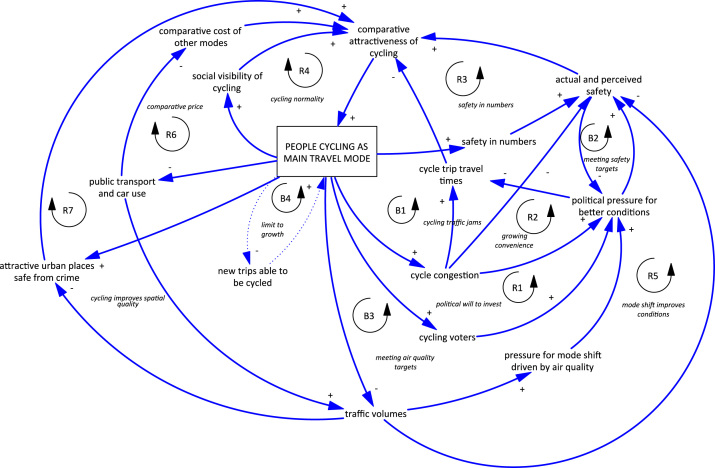
Causal loop diagram for cycling in Dutch cities.

The stakeholders’ understanding of how feedback loops create trends in cycling in the Netherlands was both more detailed and more focused on the comparative attractiveness of different modes, particularly comparing cycling with car use or travel by bus. These feedbacks are shown in Fig. 4 and described below. Of note, the initial balancing loop relating to bicycling injuries identified in both Auckland and London, was not a feature of the Dutch CLD.

Firstly, a number of helpful reinforcing loops were identified:

*R1 political will to invest*. This is very similar to the *R1* loops identified in Auckland and London. More people using bicycling as their main mode of travel creates political pressure to invest in better conditions. This improves actual and perceived safety and the comparative attractiveness of cycling.

*R2 growing convenience.* Increasing congestion on existing cycling facilities with growing numbers of cyclists also keeps the political pressure on to improve conditions, with a focus on convenience and reducing bicycle trip travel times as well as safety, also adding to the relative attractiveness of cycling.

*R3 safety in numbers.* Similar to London and Auckland, participants identified that in Dutch cities, the more people who cycled, the safer it is through a “safety in numbers” mechanism.

*R4 cycling normality.* This is again very similar to the reinforcing social normality loops identified previously.

*R5 mode shift improves conditions.* This is the same loop identified in Auckland (R4 *mode shift reduces collisions*). A significant shift from car use to bicycling can reduce traffic volumes, thereby improving actual and perceived safety and encouraging further mode shift.

*R6 comparative price.* Stakeholders considered that in the Netherlands, as more people cycled, the resulting fewer public transport passengers and reduced light vehicle numbers may mean that government revenues from these modes have declined, increasing the cost of these modes and making bicycling even more attractive as a low-cost alternative.

*R7 cycling improves spatial quality.* Finally, participants identified the importance of bicycling in “place-making”. When there are fewer cars and more people cycling, people can feel safer on the street, and public spaces become more attractive. This, in turn, makes cycling in those spaces more attractive.

There were also a number of important balancing loops identified for Dutch cities:

*B1 cycling traffic jams.* Increasing cycle congestion on many urban routes reduces the attractiveness of cycling via two mechanisms: firstly, by increasing travel times and making cycling seem less convenient; and secondly, by making cycling feel less safe, especially for parents allowing their children to cycle.

*B2 meeting safety targets.* As government cycling safety targets are met through improved infrastructure, reduced speed and lower traffic volumes, political pressure to continue improving cycling is reduced. A lack of focus on continued improvement can mean that actual and perceived safety worsens again, indeed there is some evidence that serious injuries have been increasing more recently in the Netherlands (SWOV Institute for Road Safety Research, 2015).

*B3 meeting air quality targets*. Similarly, as traffic volumes reduce, government targets to improve urban air quality are met and there is less political pressure to continue to encourage a mode shift away from car use in cities by improving cycling conditions.

*B4 limit to growth.* There was some discussion among participants about whether Dutch cities were starting to reach “peak cycling”, and that further mode shift would be pushing into longer trips and older age groups, requiring new thinking about bicycles and facilities. There was a perception among participants that commute distances in the Netherlands were increasing, limiting the number of people who could cycle as their main commute mode. This perception is supported by evidence about commute distances (Thomas and Tutert, 2013) and their impact on commuter cycling in the Netherlands (Heinen et al., 2013).

## 4 Discussion

### 4.1 Policy insights for different points on a “trajectory” of cycling

Building on previous work, we have used participatory system dynamics modelling to understand urban cycling as a complex system, including the determinants of trends in cycling in high-income cities. We have compared dynamic causal theories about cycling in three different cities, at different points on what could be considered a trajectory of cycling, from the extremely low oscillating levels seen in Auckland, to growing cycling (from a very low base) in London, to a recent history of exponential growth in cycling with now perhaps some flattening off seen in cities of the Netherlands.

The similarities in the causal loop diagrams are useful for understanding the generalizability of a causal theory about cycling across cities, at least in high-income Western cities. The three diagrams have some surprising common elements, although the words that were used by stakeholders varied. The reinforcing nature of growing numbers of people cycling for transport lifts the political will to take action to improve infrastructure, with prevention of cycling injuries and deaths being the main focus for political intervention. The reinforcing loop describing cycling safety in numbers was also a concept considered important in all three cities, as was the reinforcing pattern of cycling normality: as cycling becomes more common across age groups, genders and ethnicities, it moves out of the realm of the unusual and becomes a part of everyday life. However, in cities with more cycling, a more nuanced understanding of how this normality loop function can be elicited.

By contrast, the limits to growth due to balancing loops change along a trajectory of cycling growth. In both Auckland and London (cities at and near the start of the trajectory), the limit to growth is primarily considered to be due to the experienced and reported danger and unpleasantness when people start cycling, as well as the media reporting of deaths, as cycling increases in the absence of very significant improvements in safety.

Some further complex limits were also described in London that begin to unpick cycling normality, in particular the view that cycling is predominantly taken up by high income young and middle-aged men in the centre of the city and that without well-designed policy interventions, unhelpful balancing loops can worsen before wide participation and safety in numbers can be initiated. On the other hand, in the Netherlands, stakeholders described the limits to growth as those of cycling congestion (causing reduced sense of safety and convenience), along with the prospect of “market saturation” being reached, a place in the near future where most trips are already being cycled that can be cycled using dominant bicycle types.

The causal loop diagram for the Netherlands also demonstrates that as the predominant safety balancing loops are weakened by improved infrastructure and “safety in numbers”, the relative convenience, cost and attractiveness of different transport modes becomes important. The scope of the causal theory then necessarily needs to widen to account for feedback interplay between cycling, public transport and light vehicle use.

By identifying a more generalizable causal loop diagram for transport cycling between cities and considering a cycling growth trajectory, we can begin to draw out a more ordered set of policy recommendations for cities at different points on the trajectory. Moving cities from very low levels of cycling to sustained growth requires weakening the safety balancing loops and strengthening the helpful reinforcing links between cyclist numbers and investment in infrastructure. This can be achieved by upfront investment in improving cycling safety through infrastructure and speed management, with less emphasis on encouragement to cycle. In the early to middle portion of the growth trajectory, continued strengthening of reinforcing loops is needed, by continuing to build good infrastructure that focuses on both safety and convenience, making cycling competitive by reducing the convenience of other modes, particularly car use, building on the normality loops and broadening appeal across genders, ethnicities and age groups. Avoiding a flattening off at the top of the trajectory then requires further thinking about the late balancing loops of cycling congestion, relative attractiveness of other modes, reducing trip distances, and “market saturation”. Policies here could include maintaining political pressure for progressive reallocation of urban road space away from motorised modes to cycling and extending the “market” through good integration of cycling with public transport, as well as through bicycle technologies such as electric bikes.

### 4.2 Strengths, limitations and future directions

Participatory SD modelling allowed us to order and represent the collective values and knowledge of a wide group of cycling stakeholders, triangulating this understanding with the causal theory evident in the research literature. These collective values and shared knowledge underpin the real process of policy-making. The participatory workshops were powerful in that they enabled a *transdisciplinary* (community, policy, academic) conversation about cycling to occur, as well as group learning and insights about potentially effective policy levers. One particularly powerful insight for cities at the low end of the trajectory is a transport policy paradox: when there are very low levels of cycling, investment in very high quality infrastructure is even more important, but low cycling is also accompanied by low levels of political will to invest. Investing in high quality infrastructure that is likely to meet the transport needs of large numbers of people may help insure against unused infrastructure that undermines political will for further investment. Planning support systems such as quality of service tools and the Propensity to Cycle Tool (Cotterill et al., 2014) can assist with guiding how and where to prioritise investment, though issues of equity by income and ethnicity are missing from these tools. This is particularly unhelpful in a transport policy context where investment is responsive to changes in demand and demand forecasting based on historical trends, rather than future transport visioning and back-casting what would be needed to reach that vision (Banister, 2005, Banister and Hickman, 2013).

Testing the causal theory across different urban contexts adds to the robustness of the originally proposed theory, while building on its ability to be generalised across cities. Although generalizability is helpful for cost- and time-effectiveness, group learning occurred during the process of developing the context-specific diagrams. A balance is therefore needed between time-consuming, context-dependent repetitions of the process and the sharing of generally applicable lessons.

There are several weaknesses to this research. We have only examined three of the four “groups” of cities postulated earlier. Extending the work to a city in the second group (e.g. Germany or Norway) would improve the robustness of our insights and enable the development of a single causal diagram for urban cycling that is at least relevant for high income European and car-dependent Western cities. Extending the research to a wider range of cities would also greatly enhance the generalizability of our causal theory, including low-middle income cities and cities in China and South-East Asia, with contrasting cycling cultures. However, emerging research about cycling in Chinese cities with high levels of cycling suggests that similar causal influences may be occurring (Li et al., 2012, Lusk et al., 2014).

The policy insights that can be drawn from a qualitative causal theory are limited in two ways: the feedback loops may be refutable by drawing on routinely collected data and best available evidence; and the simulation of complex systems such as this often reveal patterns of behaviour and policy insights that could not have been predicted by qualitative analysis. The Auckland causal diagram has previously been developed into a simulation model using the best available data. Further simulation in the cities studied would assist with refining the causal theory and drawing out policy insights.
