# Supplementary material for: Understanding bicycling in cities using system dynamics modelling
Source: J Transp Health. 2017 Dec;7(Pt B):269–79. doi: 10.1016/j.jth.2017.08.002 (PMC5736169; doi:10.1016/j.jth.2017.08.002)
Supplement: Supplementary file 1 — Supplementary material [file mmc1.pdf]

Understanding London cycling trends 2013

System structure and behaviour using system dynamics modelling

Abstract

Urban cycling has the potential to bring multiple benefits for health, environment and travel choice. Increasing cycling is a policy goal for London. However, as with other transport policies, decisions about cycling are made in the context of a complex urban system, with conflicting stakeholder goals and expectations. Policies in such systems often result in unintended consequences and failure to achieve their objectives. Participatory system dynamics modelling is a method that can help with policy formulation in complex systems. In this report we lay out a series of causal loop diagrams that are intended to represent important aspects of what is and could be happening with cycling in London. These diagrams will form the basis of a computer simulation model that will allow us to test the impact of different policies over time on different outcomes. The work in developing these diagrams builds on a model developed for Auckland, New Zealand and work with stakeholders in London.

Contents

Background 2
Development and refinement of the cycling Causal Loop Diagrams (CLDs) 4
Reading the causal maps 4
Overview of the causal maps 5
Experienced and reported danger 7
Advocacy, knowledge, and effective investment 9
Normalisation of cycling 11
Stigmatisation of cycling 13
Safety in numbers 15
Data sources: Changes over time 17
Next steps 17
Acknowledgements 17
References 19

Forward:

Cycling in London is changing and the aim of this project is to use system dynamics modelling to help support the many people trying in different ways to make London a true cycling city.

Thank you to all the participants and facilitators at the London modelling workshop in May 22013 and to those who made suggestions at other opportunities. Your contributions are an integral part of producing a good model and we hope that the process of model development provides an opportunity for collaborative learning. And thank you to the LCC Policy Forum, LSHTM Transport and Health Group, and London Cycling Research group for sponsoring the workshop.

This report lays out the causal loop diagrams as they have been developed since the workshop. We have incorporated insights from the workshop and indicated key disagreements and uncertainties.

We are now hoping for further feedback on the development of the diagrams, evidence on the links (either from academic or practitioner literature), and suggestions for data on changes in variables in the models over time (both 'soft' and 'hard' variables).

We will use this data with the causal loop diagrams to create a simulation model to try and represent what has been, and what might be expected to happen with cycling in London, and to look at how different policies might help achieved desired futures.

Understanding trends in cycling in London

System structure and behaviour using system dynamics modelling

Background

Urban cycling has the potential to bring multiple benefits including improving population health, well-being, and reducing greenhouse gas emissions. Growing recognition of these benefits has led to debate about the most effective policies for increasing cycling. London has achieved some success, with cycling having doubled in the past 10 years. The Mayor has set targets for a further increase to a 5% modal share by 2026, with the GLA Transport Committee calling for a more ambitious target of 10%. However, perception of safety is the major barrier to new and increased cycling[1]. As cycling has gone up so have injuries[a 2 3]. Furthermore, it appears that the absolute risk for cyclists of serious injuries has not fallen since 2004, while cyclists make up an increasing proportion of road traffic deaths and injuries (KSI)[4]. Increasing cycling may therefore make it more difficult to meet the targeted 40% reduction in total KSI by 2040[4]. Moreover, growth in London cycling has been spatially and demographically uneven. Much of the increase has been seen in inner London, particularly in commuting from inner to central London. The people who have taken up cycling are more likely to be male, younger to middle aged, higher income and white[5].

Figure 1 shows recent and potential future trends in London's cycling mode share; one strategically desired and one "feared". In the desired future cycling meets the Mayor's target, but in the feared future cycling remains at low levels.

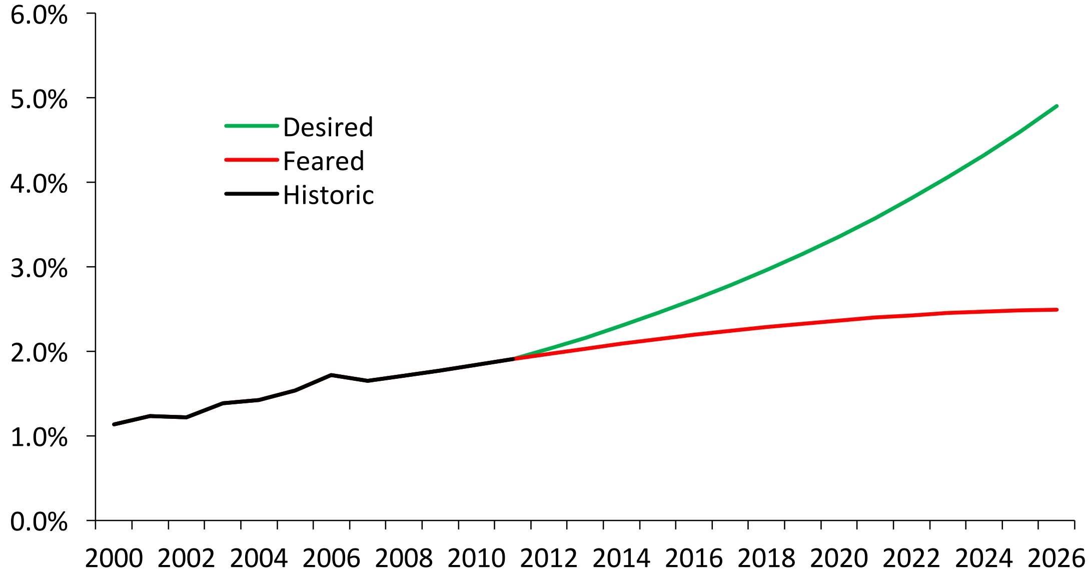

Figure 1 Desired and feared trends in London cycling mode share

Figure 2 shows recent trends and possible futures for KSI amongst cyclists and total KSI for London. The desired future here is for KSI amongst cyclists to stabilise and for total KSI to fall. The feared future is that KSI rise rapidly amongst cyclists, meaning that targets for reductions in total KSI to be missed. Achieving desired futures for both cycling mode share and KSI will require a step-change in the increase in cycling, accompanied by substantial reductions in the risks cyclists face.

[a] The picture on fatalities is less clear than on injuries, partly due to smaller numbers. Cycling fatalities appear to have remained roughly constant. As cycling has increased this would suggest risk has fallen. However, relatively risk may not have improved as deaths for other mode users have fallen.

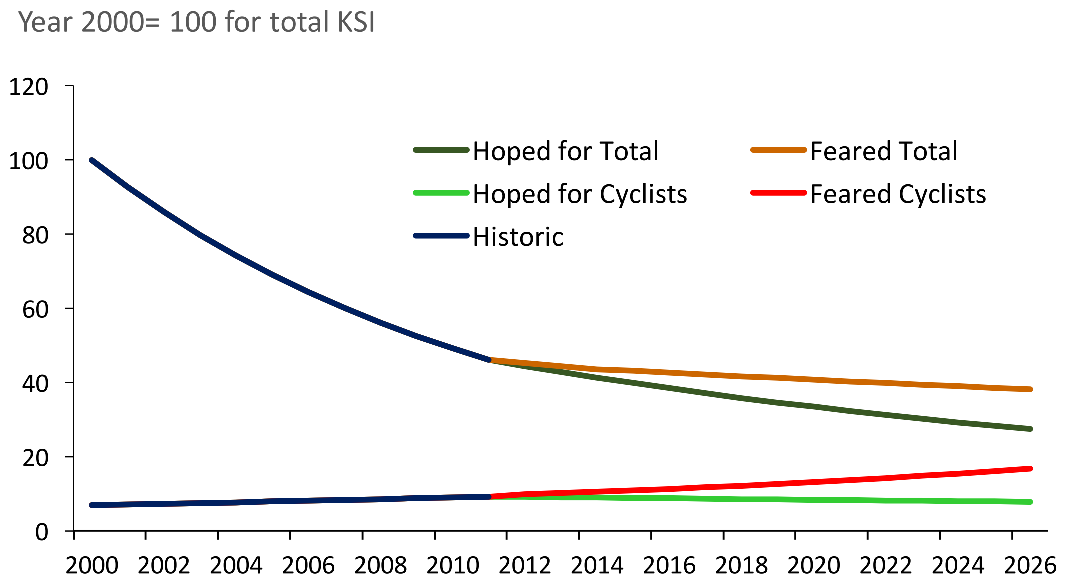

Figure 2 Desired and feared trends in London KSIs (killed and serious injuries) smoothed representation of real trends.

Greater recognition of these challenges has led to advances in the policy response (for example *The Mayor's Vision for Cycling in London*). However, disagreement remains between and within stakeholder groups about the policy priorities for achieving long-term growth. As with other transport policies, decisions about cycling are made in the context of a complex urban system, with conflicting stakeholder goals and expectations. Policies in such systems often result in unintended consequences and failure to achieve their objectives. An example of potential unintended consequences and policy failure for cycling in London might be that policies encourage cycling, but at a cost of cycling deaths and injuries, which then undermines further growth in cycling.

To address these challenges, processes are required that strengthen decision-making to:

1. Incorporate the knowledge of different groups in a participatory learning process
2. Understand feedbacks, time delays and non-linear relationships influencing trends over time
3. Explore the effects of decisions on diverse outcomes (e.g. health, environment, economy)

One method that can help meet these requirements is system dynamics modelling (SDM). When undertaken with the participation of stakeholders across policy, research and the community, SDM can help build consensus about the causal structure of the system. Disagreement and uncertainty about the feedback structure can be captured in causal loop diagrams (CLDs). Based on these CLDs and incorporating the best available data, the system can be simulated enabling competing theories to be tested, CLDs to be clarified and effective policy levers to be identified. The strengths and weaknesses of policy options over time on diverse outcomes can be compared and understood in the context of the causal diagrams. In this way, iterations of causal loop refinement and simulation continue to improve both the modelling and policy decisions. SDM has been used to support decision-making in a wide range of disciplines, including commerce, urban planning, environmental science and public health[6-10].

Development and refinement of the cycling Causal Loop Diagrams (CLDs)

A project to understand and simulate policies to increase cycle commuting in Auckland, New Zealand, was used as the starting point for discussions and workshops about cycling in London. A series of 16 in-depth interviews with a wide range of policy, community, health and academic stakeholders was followed by two workshops and further discussions with organisations and individuals. By this means a conceptual understanding was developed of the feedbacks governing commuting patterns and human wellbeing in Auckland. There were nine different parts to this initial understanding, covering aspects of land use; societal time pressures; neighbourhoods; congestion; the quality of public transport; the actions of workplaces; walking and cycling safety; environmental awareness; and the connection between car use and culture. The cycling aspects of this initial mapping of the system were drawn out to develop a simulation model and a set of realistic policy options were simulated. In Auckland, feedback loops about cycling injury and perception of safety dominated, while aspects of cycling normality, advocacy and safety in numbers were weaker in the context of very low levels of cycling and a relatively high cyclist injury rate.

We have used the cycling feedback loops from Auckland as the basis for meetings and group discussions with London policy makers, academics and advocacy organisations to develop a set of London-specific feedback loops. Initial discussions indicated that potentially nuanced feedbacks were occurring in London relating to cycling normality and advocacy for investment. A workshop was also held with stakeholders to specifically discuss cycling safety in numbers (6 March 2013, 15 participants). Through this process a set of preliminary CLDs for London were developed. These were reviewed at a single workshop held in May 2013. Thirty-two people attended the May workshop (20 men and 12 women). Present were 12 people who identified themselves as cycling advocates; 4 policy makers across health and transport; 10 academics working in public health, transport and policy studies; 5 transport engineers and planners working as consultants; and 1 NHS manager.

Participants were divided into small groups, mixing genders and roles. Groups rotated around tables, each with part of the overall preliminary system. A facilitator took each group through the part of the system on their table, encouraging participants to debate, discuss and edit the feedbacks.

Refinements were made to the preliminary feedbacks reflecting the comments and debate in the workshop, as well as triangulating the data from the workshops and discussions with the multidisciplinary literatures about cycling. Unresolved areas of debate and conflicting theories of causality are shown in these refined loops.

- **The refined CLDs are presented in this report, accompanied by a brief explanation.**
- **Areas of debate and conflict are highlighted. Suggestions for evidence that can help to resolve these uncertain or other ways to improve the diagrams are most welcome.**
- **London data on variables over time will be crucial for resolving some of the conflicting theories, as well as for simulating the model. However, other evidence can come from outside London.**
- **Data needs are therefore highlighted at the end, in the hope that you might contribute to further refinement and simulation.**

Reading the causal maps

Underlying the development of the maps are some principles about complex systems:

1. Complex systems like cycling include many interacting variables that change over time
2. It is this pattern of interaction that drives of system behaviour over time
3. Interaction between variables is characterised by feedback loops

4. Accumulation of "stocks" is also important, including people, information, or resources
5. Time matters. The pattern of cause and effect may change variables at different rates over time, creating tensions between short- and long-term policy effects

There are two kinds of feedback loop: reinforcing loops (r), so named because over time they reinforce patterns of system behaviour; and balancing loops (b) that dampen and limit trends over time. Figure 3 shows a reinforcing loop on the left and a balancing loop on the right. On the left, the more savings in the bank, the more interest is earned, reinforcing the growth in savings over time. The arrows both have +ve signs, because a change in the variable at the tail of the arrow leads to a change in the variable at the head **in the same direction**. In the balancing loop on the right, greater tiredness leads to more coffee drinking, which in turn leads to less tiredness. One of the arrows has a –ve sign because a change in the variable at the tail leads to a change in the variable at the head **in the opposite direction**. A delay between drinking coffee and feeling less tired is shown as a double line through the arrow. These delays can influence trends over time, feeling less tired may not occur immediately so that too much coffee may be drunk, overshooting the desired level of wakefulness. Combinations of loops and their relative strength explain patterns of behaviour in complex systems over time (for example in the graph).

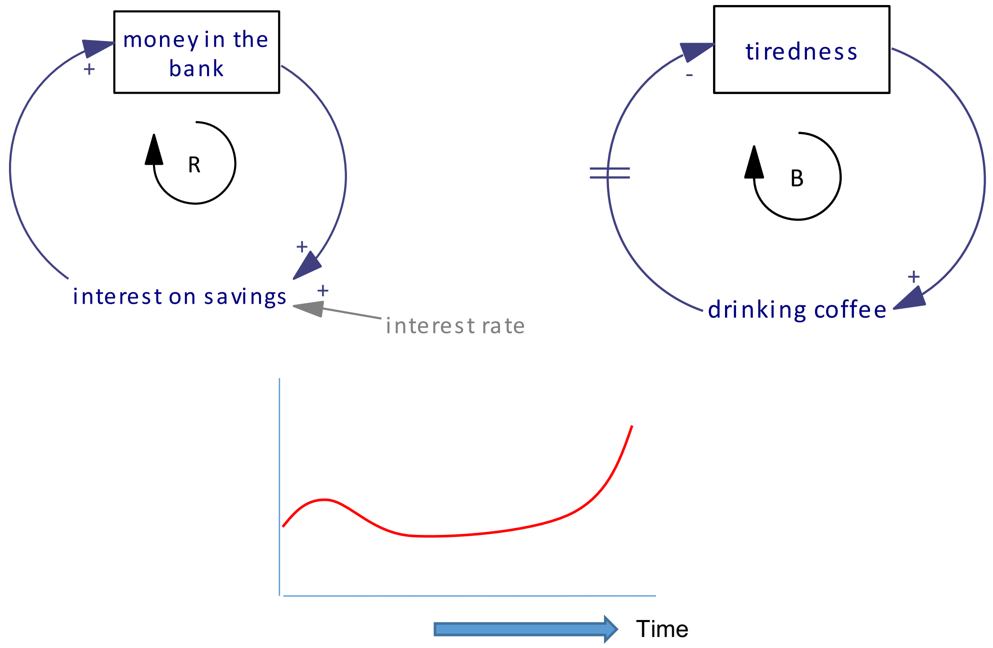

Figure 3 Reinforcing and balancing loops

Overview of the causal maps

The cycling for transport in London model has been broken up into five causal loop diagrams (sectors) for simplicity:

1. Experienced and reported cycling deaths and serious injuries
2. Advocacy and effective investment
3. Normalisation of cycling
4. Stigmatisation of cycling
5. Safety in numbers

The following high level diagram (Figure 4) summarises how these five sectors fit together. As can be seen in the overview, they are all closely interconnected.

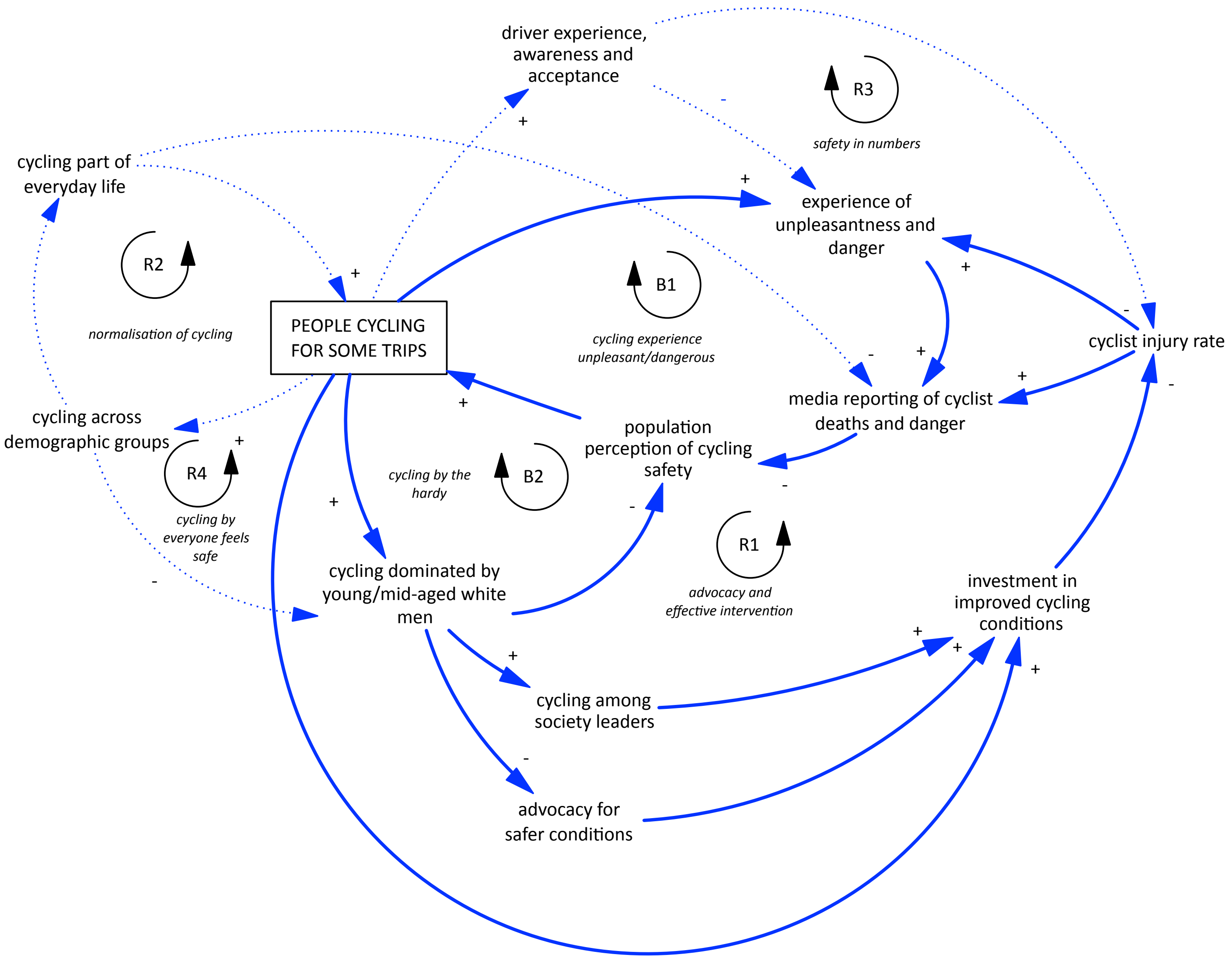

Figure 4 Overview of cycling in London

Experienced and reported danger

The effect of this sector is to dampen growth in cycling, as balancing loops dominate. Two potentially helpful reinforcing loops were considered possible but probably not currently active. The nine proposed loops are described below, and in Figure 5:

b1 media focus on deaths deters: Unless the injury rate is falling faster than the number of cyclists is growing, there will be more cycling injuries and deaths as cycling increases. Whilst the number of road fatalities for most modes has fallen substantially that of cyclists has not. In London the death of a cyclist on the road is much more likely to be reported in the media that it would have been 15 years ago[2]. More media coverage of cycling deaths may well lower the population perception of cycling safety, as well as increasing quitting by both new and experienced cyclists, acting as a barrier to further cycling growth. Some deaths may also be more 'newsworthy' than others (age, gender, location & social position), and cycling amongst more 'newsworthy' groups might have increased (see Normalisation diagram). An alternative suggested by stakeholders was *b2: cyclist cautiousness.* In this sub-loop, increased media reporting of cyclists' deaths may prompt cyclists to change their riding style and/or avoid the most dangerous routes. This could in turn lead to fewer cyclists killed or seriously injured, reducing reports in the media and so lessening the effect of b1.

b3 negative experience leads to active discouragement: The early experience of new cyclists was considered important, and there was some debate among stakeholders as to whether this was currently helpful or undermining for increasing cycling. On the one hand (and this was considered a bit more likely), a negative initial experience of cycling due to heavy and fast traffic, poor quality infrastructure, or personal injury was likely to lead to early quitting, and beyond this to spreading the word that cycling was dangerous. However, an early positive experience could help with enthusiastic word of mouth "marketing" of cycling (*r2 new enthusiasts*).

b4 "trending" is news: it's possible that what makes cyclist deaths newsworthy is that cycling was unusual, but is now rapidly increasing among some groups of people – a trend stimulating media interest. However, if cycling increased to levels that made it seem a normal part of everyday life (like travelling by car or walking) then potentially cyclist deaths would be less of a story (*r1 no story in the everyday*). Reduced coverage would then improve population perception of safety.

b5 more injuries discourage policy makers: If cycling comes with a higher risk than other modes then encouraging cycling may conflict with policy safety targets. As cycling injuries increase this may therefore lead to reduced policy commitment to cycling. A reinforcing loop is also likely with increasing political will to improve conditions for cycling (see *Advocacy and infrastructure loops*). Similarly, the strategic promotion of cycling creates greater media scrutiny and responsibility of policy makers for cycling deaths (*b6 encouragement has consequences*).

b7 safety campaigns make people nervous: Finally, cycling safety campaigns in the media that focus on cyclists' behaviour as a response to reported deaths were considered to reduce population perception of safety and therefore be counter-productive by many stakeholders.

A number of other exogenous variables (not involved in feedbacks) were considered important influences on cyclists quitting. These included sense of personal security (particularly for women outside of daylight hours and teenage boys in particular localities) and bicycle theft.

[2] Preliminary analysis led by Dr Anna Goodman, anna.goodman@lshtm.ac.uk

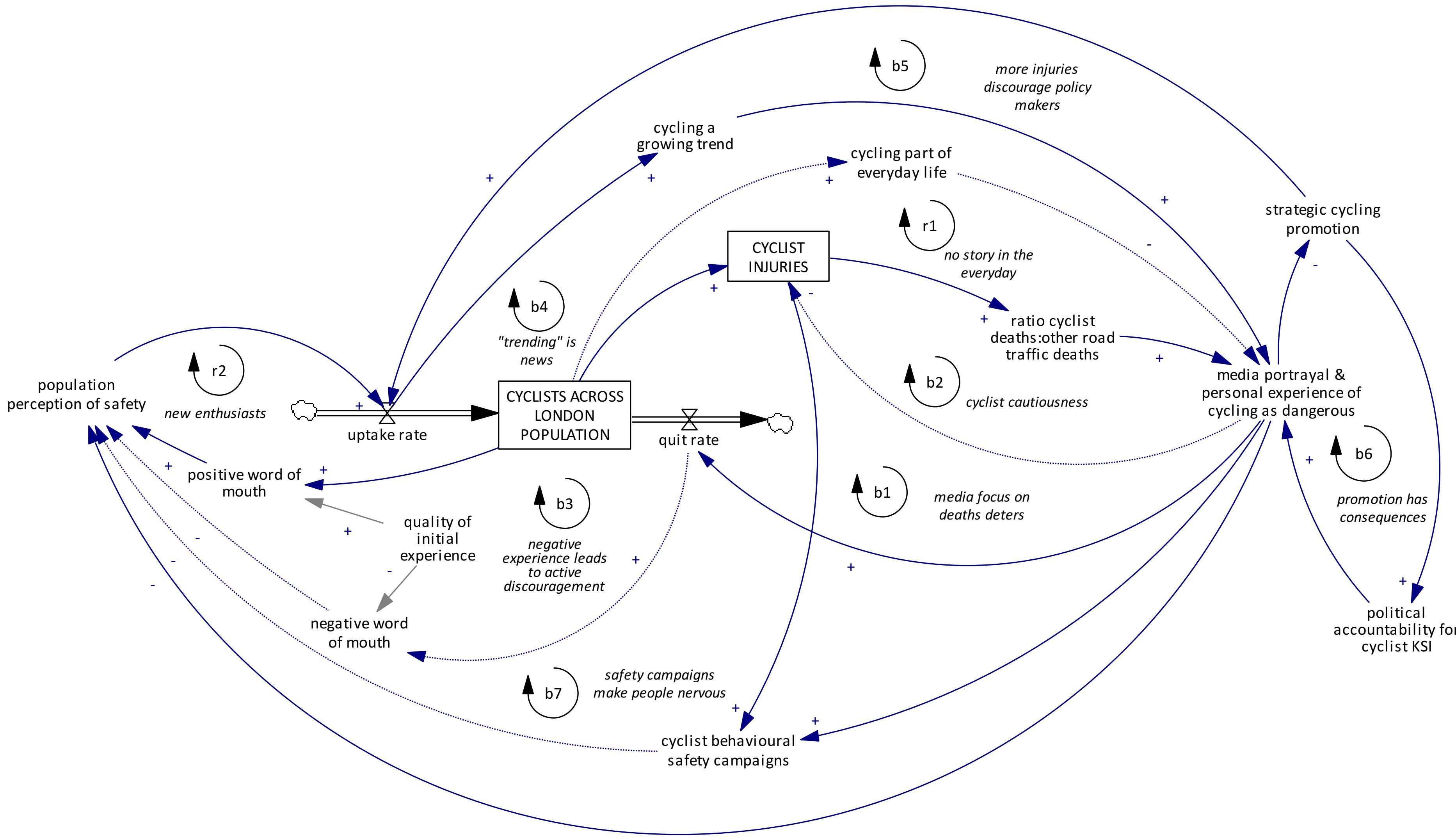

Figure 5 Experienced & reported danger

Advocacy, knowledge, and effective investment

This sector is dominated by potentially competing reinforcing loops, many of which are considered to be active in London currently. There are six reinforcing loops and two balancing loops.

r1 visible spend initially encourages more cycling: an increase in advocacy about cycling safety and infrastructure, prompted by intense media reporting of cyclist deaths and serious injuries, leads to increased political will to intervene, and pressure to "just do something". The consequent actions initially increase cycling even if the interventions are ineffective for at improving safety. However, the increase in cycling would be temporary as more people recognised the ineffectiveness of interventions.

This "do something" spend produces ineffective interventions that do not improve either actual or perceived safety. In turn this would lead to further activism in response to further cyclists seriously injured or killed- particularly if warnings were not heeded at previously identified dangerous locations. *r2 'do something' spend leads to ignored warnings.* However, in the longer term there may be a balancing loop if poor infrastructure continued to be built then over time *b1 poor investment leads to disillusionment* and advocacy would fall.

On the other hand, with some delays, over time institutional learning occurs (both in direct response to political will and advocacy – *r3 effective spend lowers risk & increases cycling, r5 – effective spend makes cycling feel safer* and with more experience of interventions – *r4 knowledge loop*), and interventions become more successful at increasing both actual and perceived safety, encouraging more cyclists. Knowledge was seen as coming from, and being embedded in, engineers, planners, senior policy makers, researchers and advocates. Views varied on how quickly these different groups would learn. Examples of good infrastructure visible in the region might continue to encourage advocates in a positive reinforcing loop, maintaining pressure to continue high quality interventions (*r6 – good examples encourage advocacy*). On the other hand, the improved safety and mode share would also decrease media reporting of cycling injuries, thereby reducing the impetus for advocacy (*b2 – good infrastructure reduces pressure to act*).

There was consensus among stakeholders that good evaluation and evidence about interventions was needed to accelerate the knowledge loop.

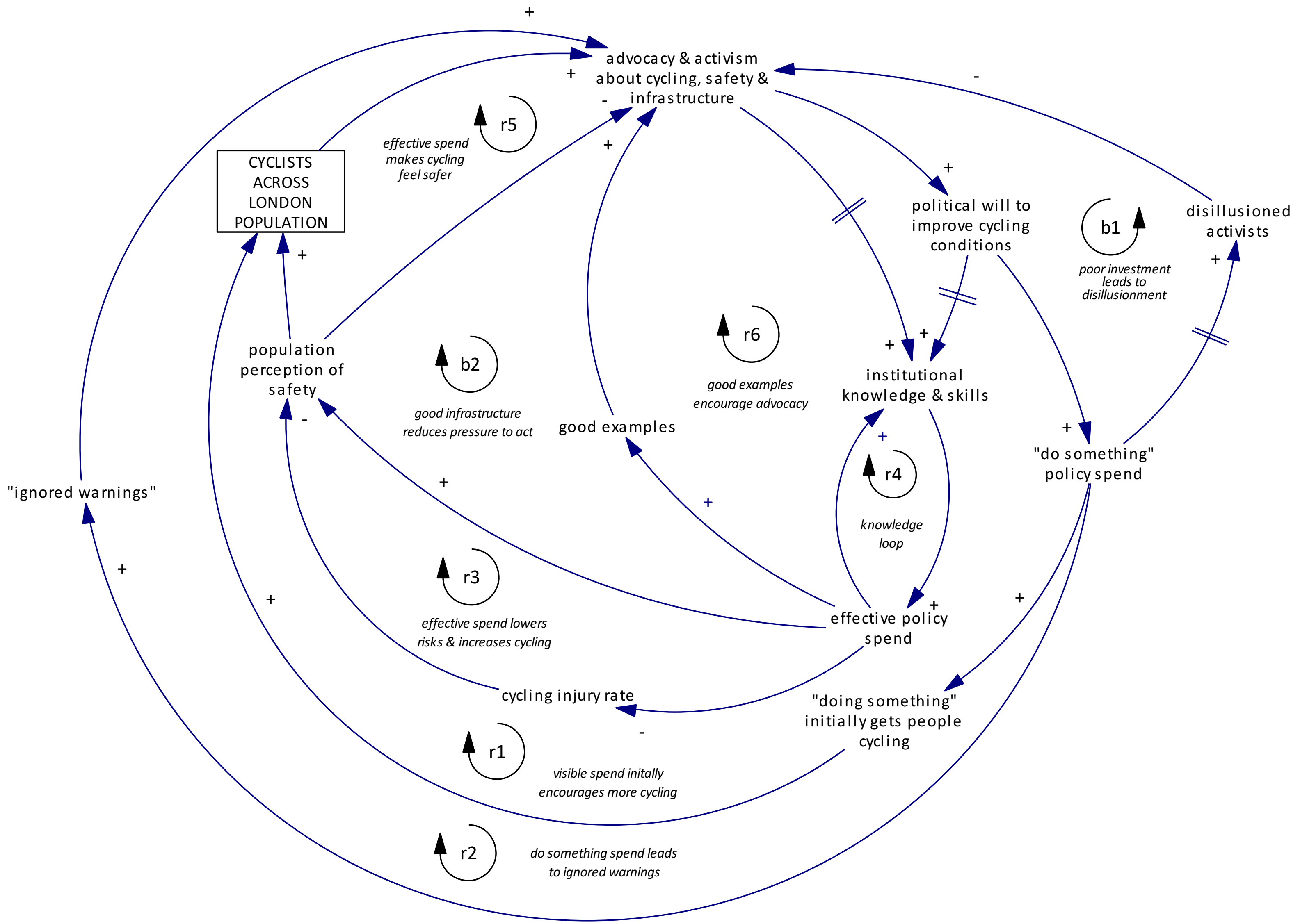

Figure 6 Advocacy, knowledge, and effective investment

Normalisation of cycling

This sector consists of a mixture of reinforcing and balancing loops (four reinforcing and two balancing) and describes the uptake of commuter cycling into central London and the way this interacts with wider urban cycling. On the whole, discussions with stakeholders suggest the helpful reinforcing loops appear currently to be acting more strongly than the balancing loops in this sector.

In *r1 cycle commuting normalised*, the growth in cycling into central London increases the population perception that cycling is a legitimate mode of commuter transport. This normalisation, perhaps slowly, increases other cycling across the population. However, cycle commuters appear to be more likely to wear specialist cycle clothing than other cyclists (perhaps because of longer journey distance) and this may limit the normalisation from more commuter cycling[11]. A similar loop *r2 cycling is an everyday activity* can happen for cycling in London more generally but this was considered to currently be less active.

r3 cycling aspirational: people who commute into central London at rush-hour and live within a reasonable cycling distance are likely to amongst the more educated and privileged. There appears to be a particularly large increase in cycling amongst these groups. This may well enhance the aspirational and fashionable profile of cycling, shifting previous cultural associations of cycling with poverty. This same educated, privileged and fashionable group also include many social leaders, opinion formers and even celebrities who can influence media representations of cycling, further enhancing the profile of cycling (*r4 cycling as legitimate & fashionable*). These leaders and opinion formers also have influence over large organisations, shifting organisational cultures towards cycling (*r5 organisational culture shift*).

Two balancing loops are also possible, but may not be currently active. *b1 road and public transport congestion:* The congestion of motorised modes (including roads, parking and public transport) can encourage more cycling. However, with a significant mode shift, cycling could relieve congestion for other modes, reducing its effect on cycling uptake, (see also *Safety in numbers* loops).

b2 cycle route congestion: Significant uptake of commuter cycling has led to congestion on popular cycle routes into central London. It may be possible that this congestion becomes a barrier to further growth in commuter cycling, although this was contentious. r1 and b2 are closely linked. r1 is the positive side of seeing lots of cyclists on a given route, whilst b2 is the negative side that past a certain point this makes cycling more difficult and minor incidents more likely.

Many of the variables in this sector lead to increased advocacy and influence on policy, particularly cycling among leaders and opinion formers, but also congestion of cycle facilities. However, there were considered to be downsides from the uptake of cycling being led by particular groups; with this potentially increasing views of cycling as exclusive and stereotyping of cyclists, thereby reducing uptake amongst other groups and feeding stigmatisation of cyclists (see Stigmatisation diagram).

A large number of exogenous factors were considered important in this sector. Cycling uptake by the educated and privileged may be increased due to their susceptibility to health messages; there are socio-economic and ethnic inequalities in commuting from inner to central London; and the success of sports cycling was thought likely to have an impact (at least in the short term). The Congestion Charge was considered important for influencing cycling at peak time in central London. Stakeholders suggested that hilliness and distance influenced whether people cycle in work clothes. Cultural factors were also considered important; including barriers (particularly for women), whilst in others already changing cultural norms around driving (particularly amongst young men) could encourage cycling.

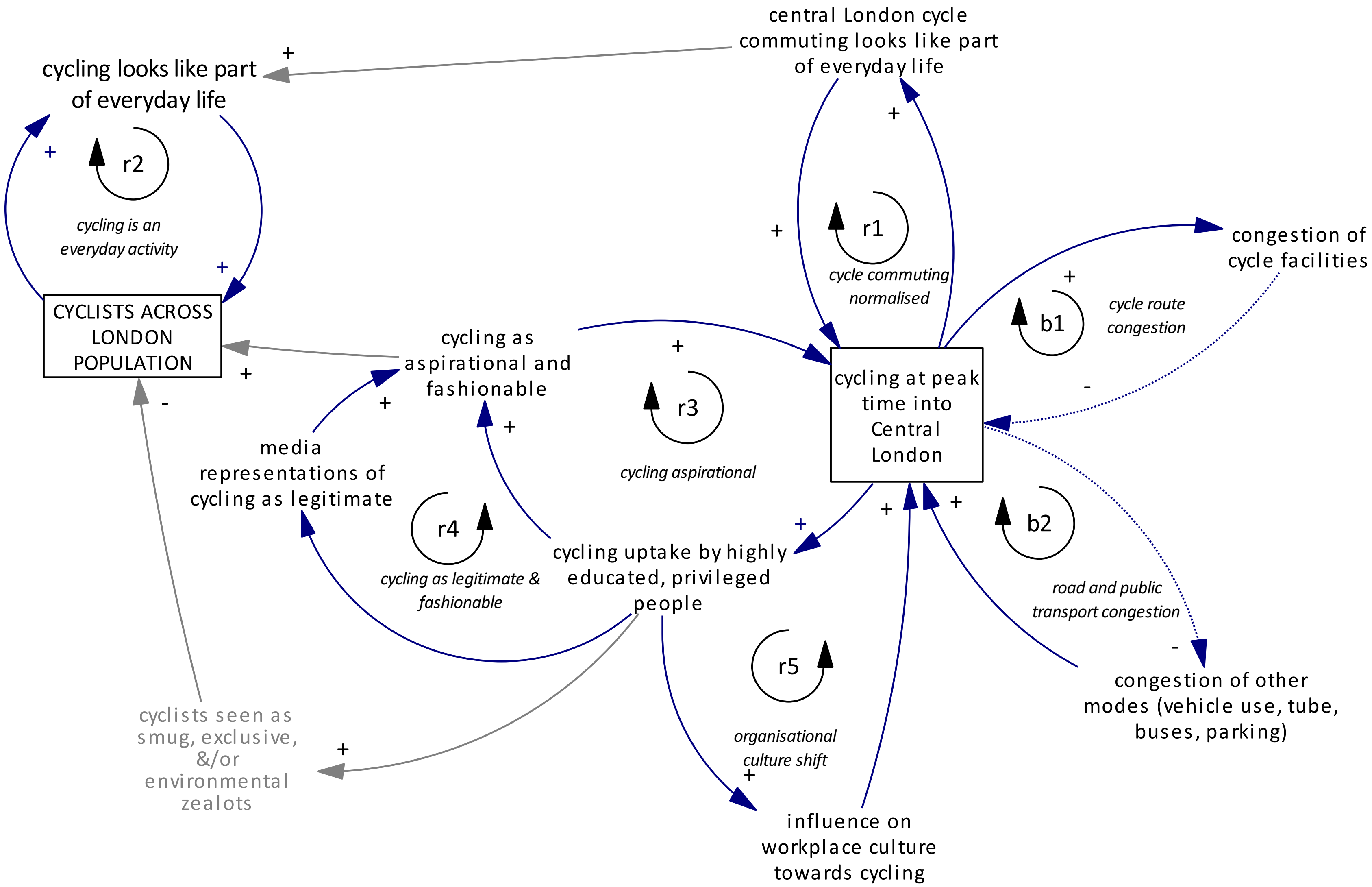

Figure 7 Cycling normalisation in Central London

Stigmatisation of cycling

This sector can be viewed as both the response to a system that privileges motorised transport, and the negative side of particular groups being more likely to stick with cycling in difficult conditions. Stigmatisation is taken to mean branding as socially negative or abnormal. The sector is dominated by unhelpful reinforcing loops. There was a good agreement among stakeholders that these loops describe London's current situation.

r1 wider uptake inhibited by poor conditions: Low perception of safety across population groups limits uptake to those willing to cycle in what are perceived to be dangerous conditions. This limited risk-tolerant group tends not to demand improved conditions (focusing more on the skills of cyclists), perpetuating low population perception of safety. They also need to be socially secure in a system where cycling's social legitimacy is low. Therefore they are likely to be not only men, but white British, since they have protection from some other kinds of discrimination (sexism or racism).

r2 (accepted role of cycling) describes the role that visible infrastructure can play in increasing the legitimacy of cycling – where actions to improve the quality of cycling conditions can increase its legitimacy, thereby increasing cross-population cycling and reducing its dominance by the risk-tolerant.

In *r3 stigmatisation through perceived risk-taking*, this particular group of risk-tolerant cyclists (young to middle-aged men) tend to be seen as risk-takers more widely in society (for example, linked to their dominance in car crash injuries, risk-taking sports and alcohol and drug use), which lends cycling a "risk-taking" stigma, reinforcing its dominance by the risk tolerant. On the other hand, a balancing loop (*b1 lower risk for the middle-aged and men?*) is that middle-aged men appear to have a lower fatality rate whilst cycling than women or older men, keeping down the number of deaths.

r4 stigmatisation through perceived rule-breaking: this loop makes a figure-of-eight. The suggestion here is that the perception of cyclists as rule breakers partly stems from the lack of place for cyclists in the built and regulatory environment and therefore low social legitimacy. This then reinforces cycling's dominance by the risk-tolerant, and the negative impact of this dominance on action to improve conditions. .

Other pathways by which cycling is stigmatised in reinforcing patterns include the way that low perception of safety encourages people to wear special safety gear (high-vis and helmets), rather than just everyday clothes. In addition, the attraction of young to middle-aged men is also seen as being related to fitness consciousness in that group, encouraging the use of special 'sporty' clothes for cycling (*r5 stigmatisation through special kit*). There was disagreement among stakeholders as to whether the dominance of transport cycling by young to middle-aged men affected cycling's perception of it being for fitness and health *r6 sport, age and gender*. The final loop, *r7 attraction of rebels* suggests that the perception of transport cyclists as risk-takers could lead to cycling continuing to be attractive to young and middle-aged men for just this reason.

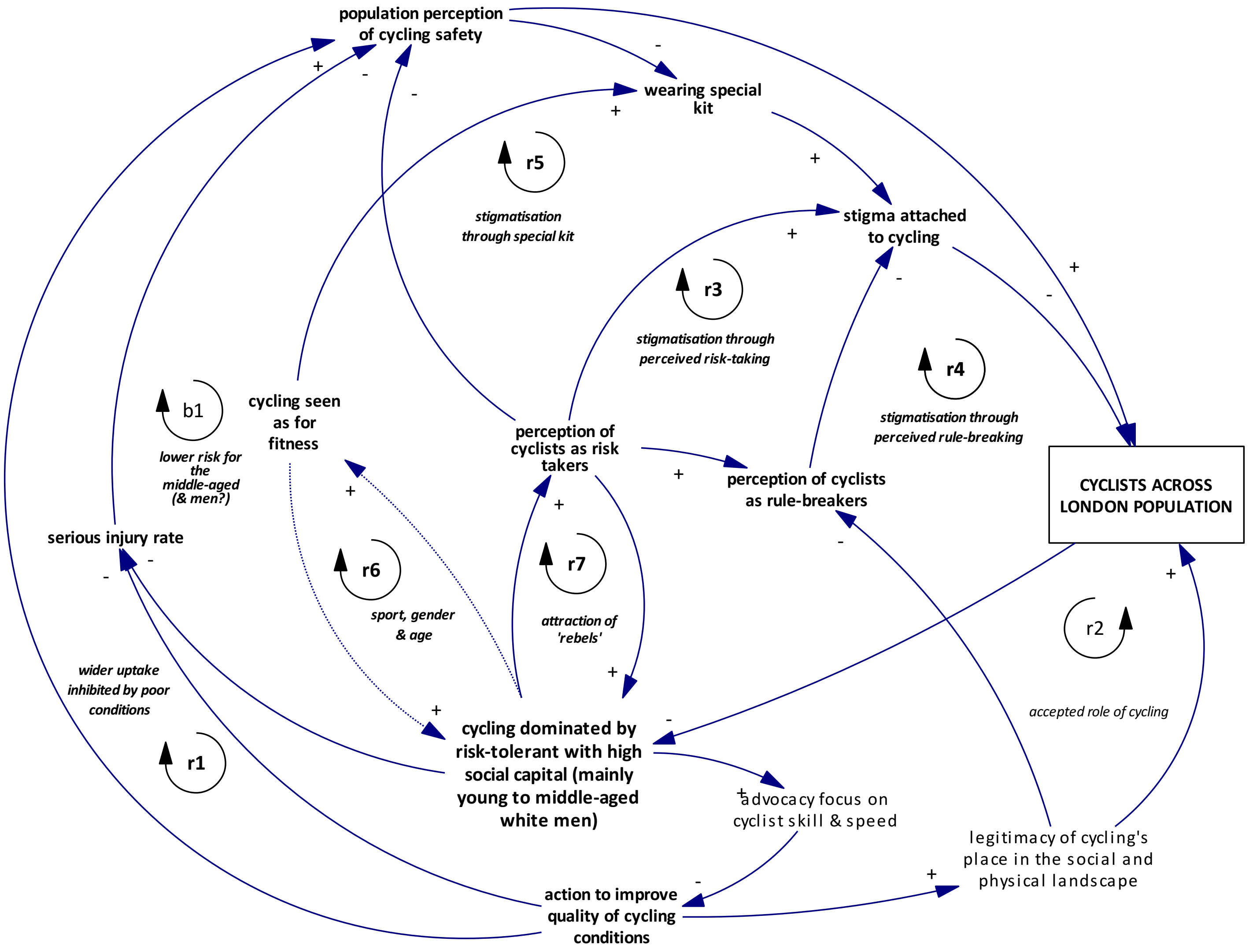

Figure 8 Pathways to cycling stigmatisation

Safety in numbers

This sector describes a set of potentially virtuous reinforcing loops that comprise what is understood as the "safety in numbers" phenomenon for cycling, plus one possible balancing loop. The ecological relationship described between increasing cyclists and reducing cycling injury and fatality rates in published studies is likely to be a result of the reinforcing loops described here.

r1 safety by design: A significant proportion of the reported safety in numbers effect in ecological studies is likely to be explained by "numbers in safety". In other words, more cyclists leads to increased quality of cycling conditions, reducing the injury rate and hence the quit rate and encouraging uptake across the population. The relationship between cyclist numbers and effective intervention has been described in more detail in the *Advocacy and effective intervention* sector. Of all the loops in this sector, this was thought by many stakeholders to be the strongest loop acting in London currently.

It's possible that a significant shift to cycling might reduce motor vehicle volumes improving both actual and perceived safety and increasing the number of cyclists (*r2 safety by mode shift*). However, these loops were not considered to be currently important in London, for two reasons. Firstly, most people would be replacing public transport trips with cycling rather than private motor vehicle trips. Secondly, reduced congestion could lead to faster speeds and then more traffic unless road space is systematically reallocated from motor vehicles to cycling as cycling increases. If more space was taken up by HGVs then cycling fatalities could actually increase.

r3 safety by awareness, knowledge & acceptance: This is more classically how safety in numbers is understood. Through a combination of increased cyclist presence and an increasing proportion of motor vehicles driven by people who either also cycle, or whose close contacts cycle, more cycling raises driver awareness and knowledge about good driving practice in the presence of cyclists. This reduces the cycling injury rate leading to more cycling. Awareness through visibility, personal contacts, and personal experience would occur at different levels of cycling uptake. There was agreement that this loop may be occurring in certain areas of inner London. Beyond awareness and knowledge, acceptance might be more important for deliberate rather than 'accidental' dangerous driving. One hypothesis is that acceptance is linked to a social or cultural mismatch between cyclists and drivers. However, like cyclists, drivers in central London are more likely to be male and of similar ages. Stigmatisation may be a stronger explanation for deliberately dangerous behaviour.

Finally, a balancing loop was also thought possible, *b1 danger in numbers*: in the context of inadequate infrastructure, congestion on cycle lanes may push cyclists into traffic situations that are more dangerous (either through using less congested but more dangerous routes, or through overcrowded cyclists getting in each other's way), creating a balancing loop by increasing injury rates, reducing perception of safety and inhibiting growth in cycling.

Variables from other loops are also important. Normalisation: Cycling among leaders and opinion formers reduces media negative stereotyping of cyclists and therefore driver animosity. Cycling among leaders and opinion formers also encourages media mainstreaming of cycling policy, increasing driver awareness and knowledge.

Exogenous variables were mentioned. The proportion of cycle trips undertaken off road increases population perception of safety and attractiveness of cycling, but also reduces cyclist presence on the road network. The public cycle hire scheme is very visible and directly increases cycling's presence in the road network. It was also suggested that more normalised and uniform behaviour by cyclists might strengthen loop r3.

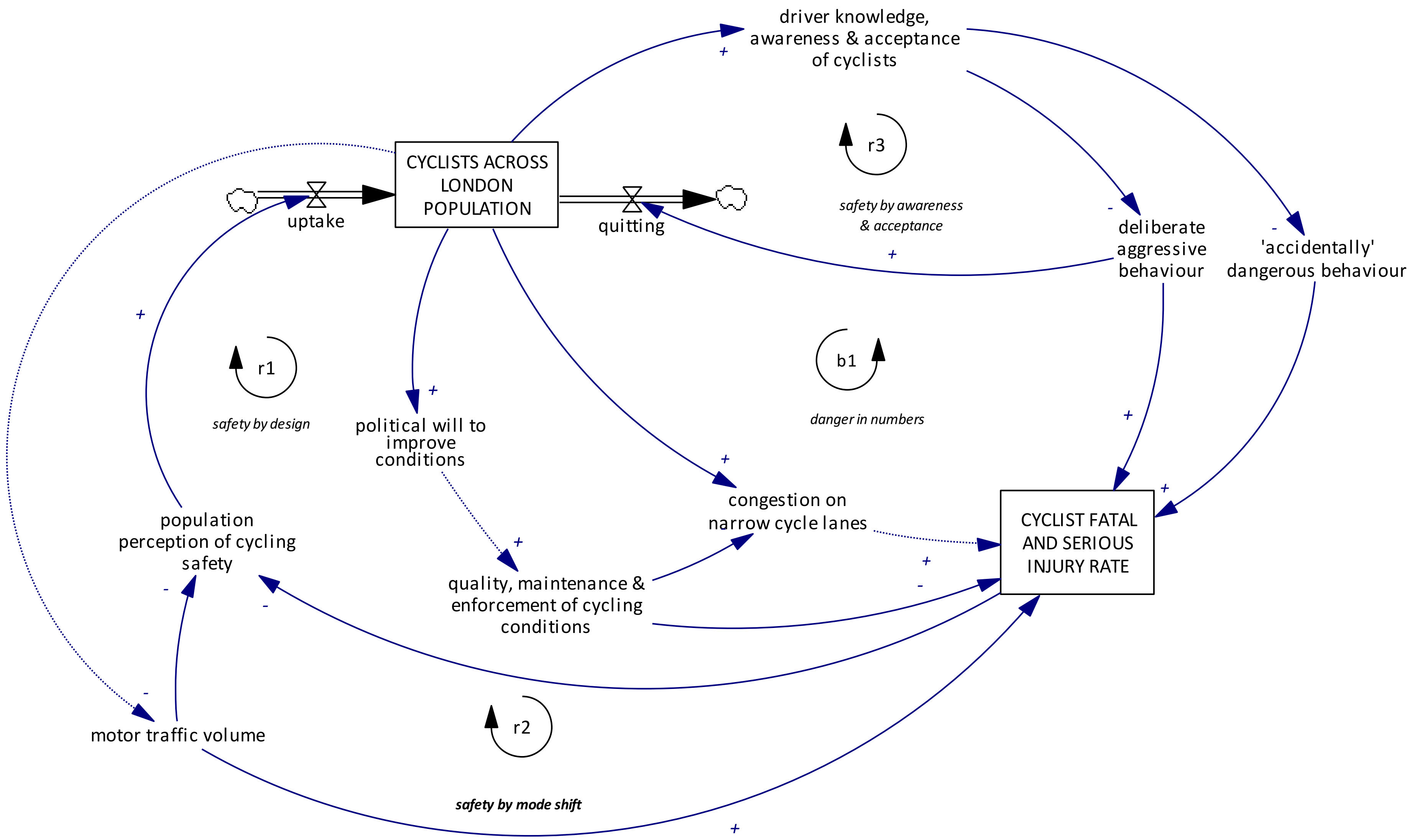

Figure 9 Safety in numbers

Data sources: Changes over time

To better understand and validate the proposed feedback loops and begin to simulate the cycling system in London, we are requesting assistance with identifying data. Based on the CLDs described above, Table 1 includes a list of data needs. Blank spaces are left for suggestions, even where some data are already available more would be better. Please feel free to cut and paste this table into an email to James Woodcock (jw745@medschl.cam.ac.uk). Ideally, much of the data would include a number of points **over time** (longitudinal).

We would also welcome suggestions on how to deal with variables for which there is insufficient data.

Next steps

The understandings generated in this process will be used to generate a policy relevant simulation model as a collaboration between CEDAR and the MRC Biostatistics Unit. For more information on the development of the model please contact James Woodcock jw745@medschl.cam.ac.uk or Chris Jackson chris.jackson@mrc-bsu.cam.ac.uk

Acknowledgements

Academic: CEDAR, UCL, & MRC

People who have assisted with the development of the London model: Rachel Aldred, Alex Stredwick, Anna Goodman, Rebecca Steinbach, Greg Cowan + all the participants at the workshop

Sponsorship of the workshop: LCC Policy Forum, London Cycling Research Group, LSHTM Transport & Health Group

Main Diagrams	*Data: Change over time*	*Possible sources*
**Multiple**	Cycling London by area, purpose, age, gender, ethnicity, income	London Travel Demand Survey (LTDS), Traffic counts
**Experienced & reported danger & Safety in numbers**	Cycling deaths and injuries by area, gender & age	Stats19
**Experienced & reported danger**	Uptake & quit rates for cycling in London	
**Experienced & reported danger**	Perception of cycling safety	British Social Attitudes survey, Attitudes to Cycling
**Experienced & reported danger**	Quantitative & qualitative data on media reports of cyclist deaths	Initial quantitative media review, follow up with qualitative analysis
**Advocacy/ Normalisation**	Other types of media reporting on cycling	
**Stigmatisation/ Experienced & reported danger**	Quantitative & qualitative data about barriers to uptake	Quant: Attitudes to Cycling<br>Qual: Research e.g. Green & Steinbach/ Understanding Walking & Cycling
**Experienced & reported danger**	Quantitative & qualitative data about reasons for quitting	Quant ?/ Qual: Research studies
**Advocacy**	Policy commitment to cycling	Qualitative analysis of policy documents & media reports?
**Advocacy**	How do we measure advocacy? Amount & demands	
**Advocacy**	Investment in interventions in London	
**Advocacy**	Quality & quantity of cycling infrastructure in London (including maintenance)	
**Advocacy**	Evidence about the effects of different interventions on actual and perceived safety, & mode share	Research studies – systematic reviews have identified limited rigorous scientific evidence
**Experienced & reported danger**	Evidence about the effectiveness of promotion	
**Advocacy**	How long does institutional learning take & how does it happen (advocacy/ staff turnover/trial-and-error/evaluation)	
**Advocacy**	Changes in advocacy in cities with different levels of cycling	
**Stigmatisation/ Normalisation**	Perceptions of cycling & cyclists in London (other than safety)	Attitudes to Cycling, research studies (e.g. Cycling Cultures)
**Advocacy**	How might we think about organisational culture shift?	
**Safety in numbers**	Cyclist-reported driver careless & aggressive driving	
**Safety in numbers**	Knowledge, skills, awareness and acceptance of cyclists by drivers (including commercial vehicles)	
**Safety in numbers**	Social mismatch between cyclists and drivers in London	LTDS for passenger travel but also need data on commercial vehicles
**Safety in numbers**	Reported injuries on congested cycling infrastructure	Stats19

Table 1 Data needs

References

1. Transport for London. Attitudes towards cycling: Annual report 2012 TfL number: 05110 v1.0. London: Mayor of London, Transport for London, 2012.
2. STATS19 Available from:http://www.stats19.org.uk/ (last accessed 9th July 2013).
3. Transport for London. Casualties in Greater London during 2012 June 2013. London: Transport for London, Mayor of London, 2013.
4. Transport for London. Safe Streets for London: The Road Safety Action Plan for London 2020 Working together, towards roads free from death and serious injury, 2013.
5. Goodman A. Walking, Cycling and Driving to Work in the English and Welsh 2011 Census: Trends, Socio-Economic Patterning and Relevance to Travel Behaviour in General. *PLoS One* 2013;8(8):e71790.
6. Videira N, Antunes P, Santos R, Lopes R. A participatory modelling approach to support integrated sustainability assessment processes. *Systems Research and Behavioral Science* 2010;27(4):446-60.
7. Goh YM, Love PED. Methodological application of system dynamics for evaluating traffic safety policy. *Safety Science* 2012;50(7):1594-605.
8. Hjorth P, Bagheri A. Navigating towards sustainable development: A system dynamics approach. *Futures* 2006;38(1):74-92.
9. Sterman JD. Learning from Evidence in a Complex World. *American journal of public health* 2006;96(3):505-14.
10. Sterman JD. *Business Dynamics Systems Thinking and Modeling for a Complex World*. Massachusetts Institute of Technology, Sloan School of Management, 2000.
11. Goodman A, Green J, Woodcock J. The role of bicycle sharing systems in normalising the image of cycling: an observational study of London cyclists. *Journal of Transport and Health* Accepted for Publication.
